# Supplementary material for: Synthesis of Platinum Nanocrystals Dispersed on Nitrogen-Doped Hierarchically Porous Carbon with Enhanced Oxygen Reduction Reaction Activity and Durability
Source: Nanomaterials (Basel). 2023 Jan 21;13(3):444. doi: 10.3390/nano13030444 (PMC9919006; doi:10.3390/nano13030444)
Supplement: Supplementary file 1 [file nanomaterials-13-00444-s001.zip › nanomaterials-2141788-supplementary.pdf]

Supporting Information

Preparation of Platinum Nanocrystals Dispersed on the Nitrogen-doped Hierarchical Porous Carbon with Strong Pt-N Interaction to Enhance Oxygen Reduction activity and durability

*Min Li, Feng Liu, Supeng Pei, Zongshang Zhou, Kai Niu, Jianbo Wu, Yongming Zhang**

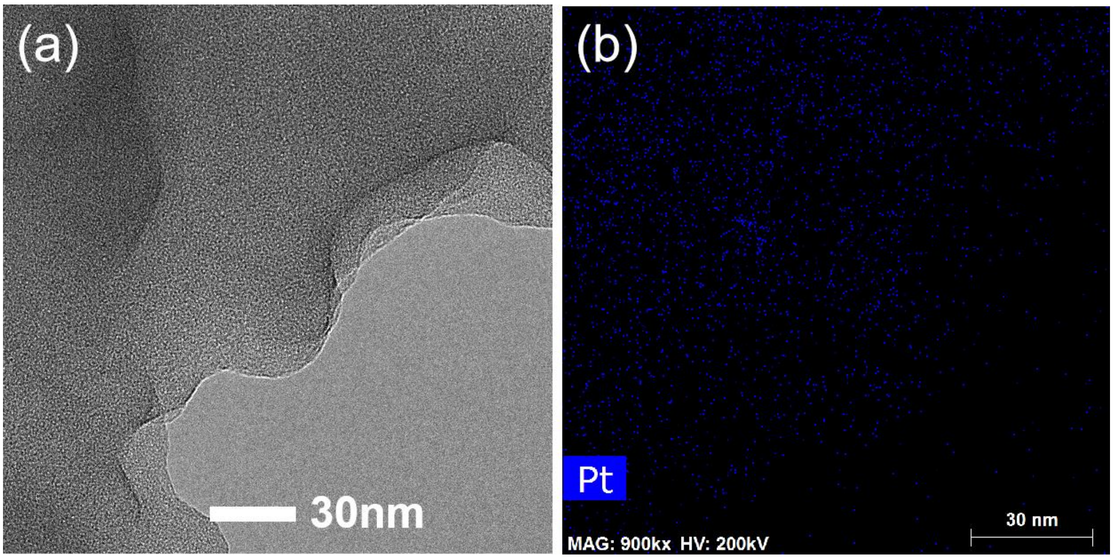

Figure S1. TEM image of Pt@Resin(a) and element map of Pt(b)

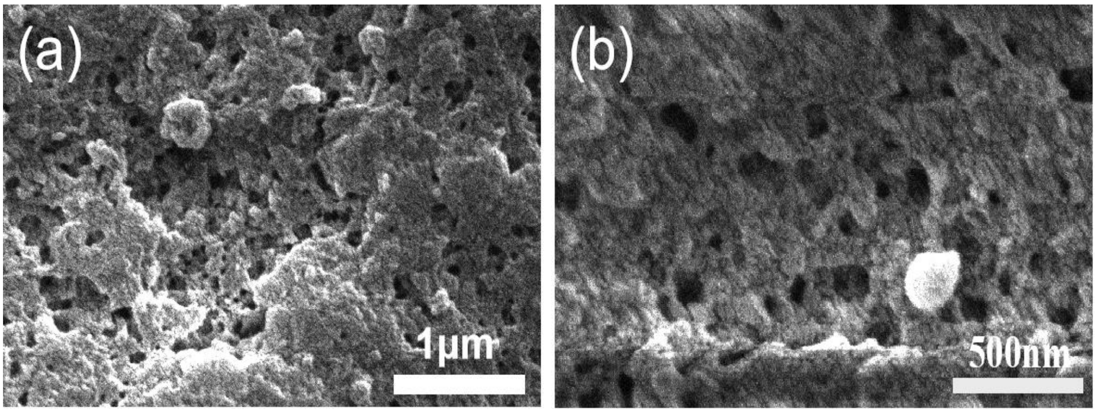

Figure S2. (a,b) SEM image of Amberlite Q-PSDB resin

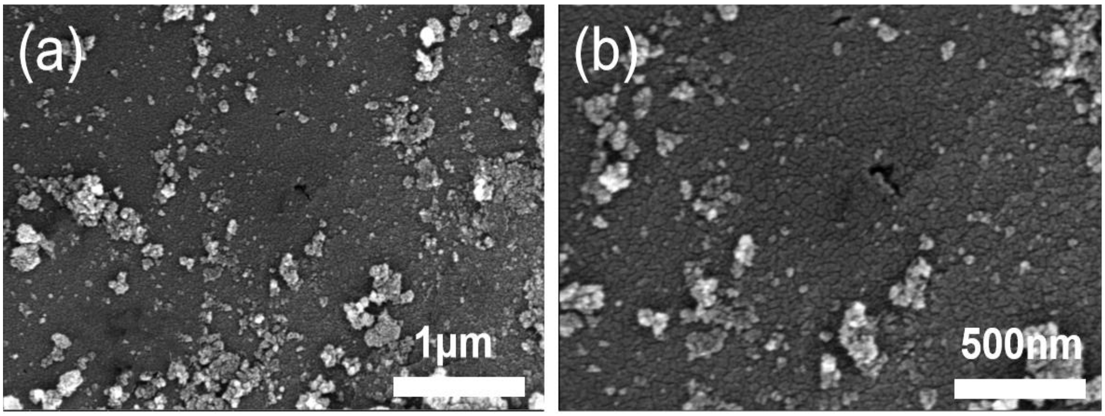

Figure S3. (a,b) SEM image of as-prepared Pt/NHPC-800

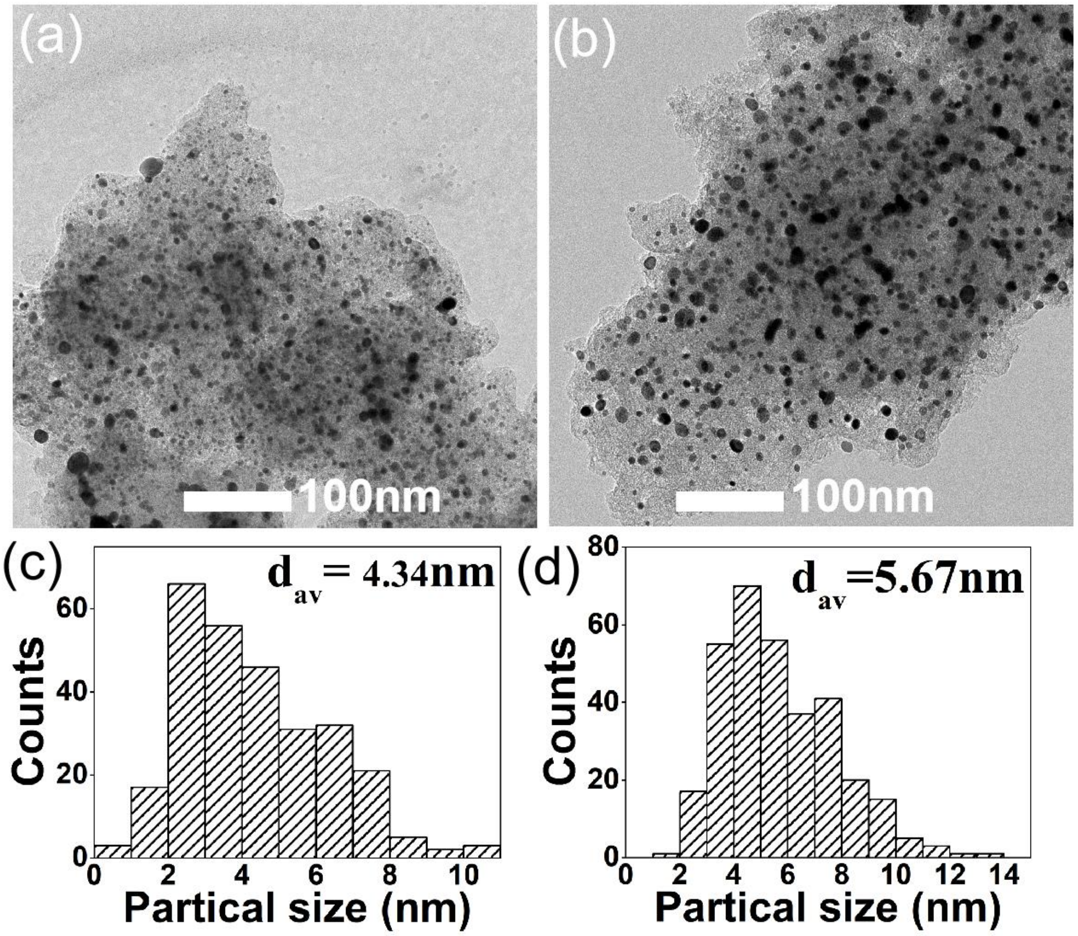

Figure S4. TEM images of the (a) Pt/NHPC-700, (b) Pt/NHPC-900 and partical size distribution of the (c) Pt/NHPC-700, (d) Pt/NHPC-900.

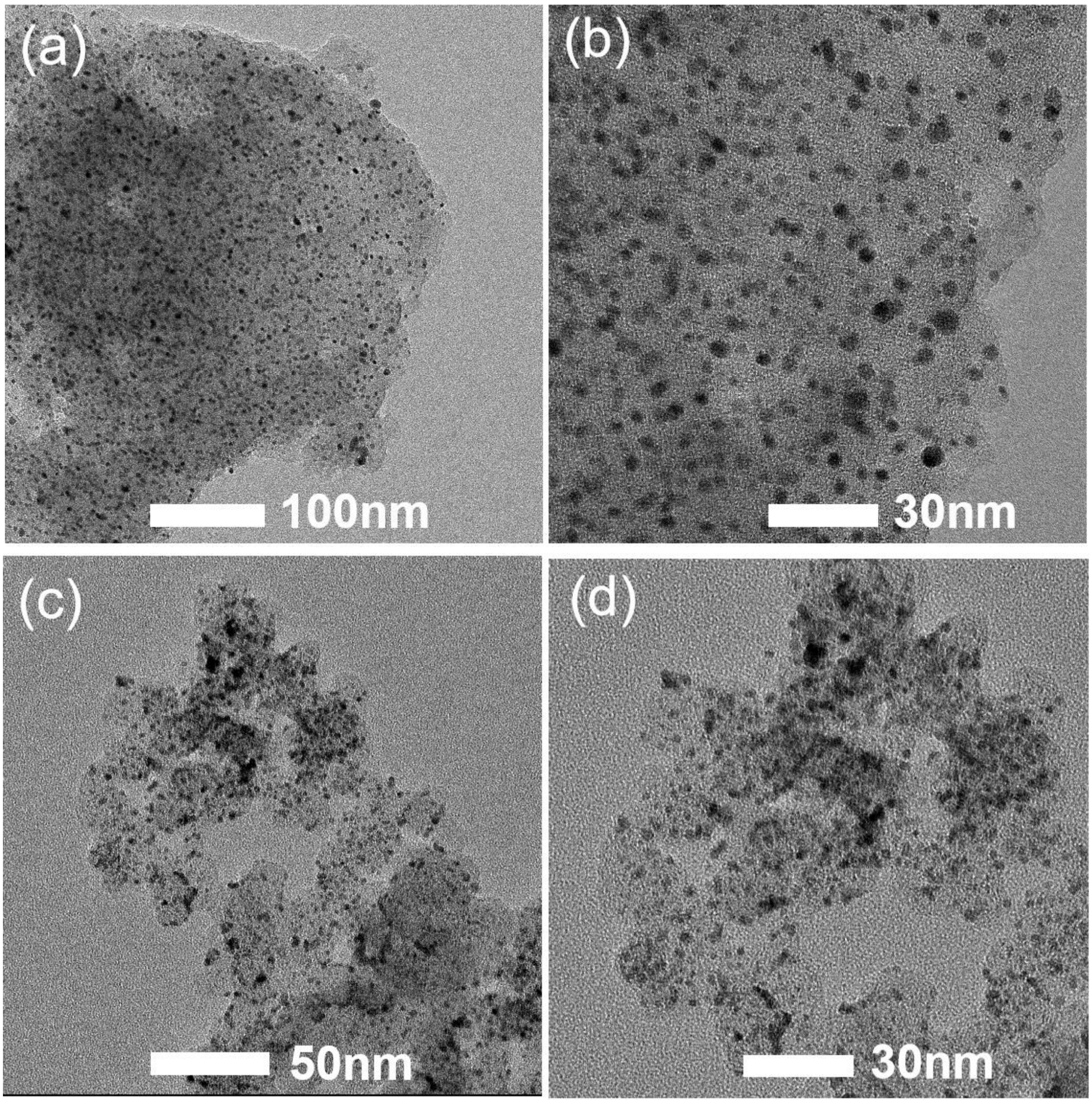

Figure S5. TEM image of as-prepared Pt/NHPC-800 (a,b) and the commercial Pt/C (c,d)

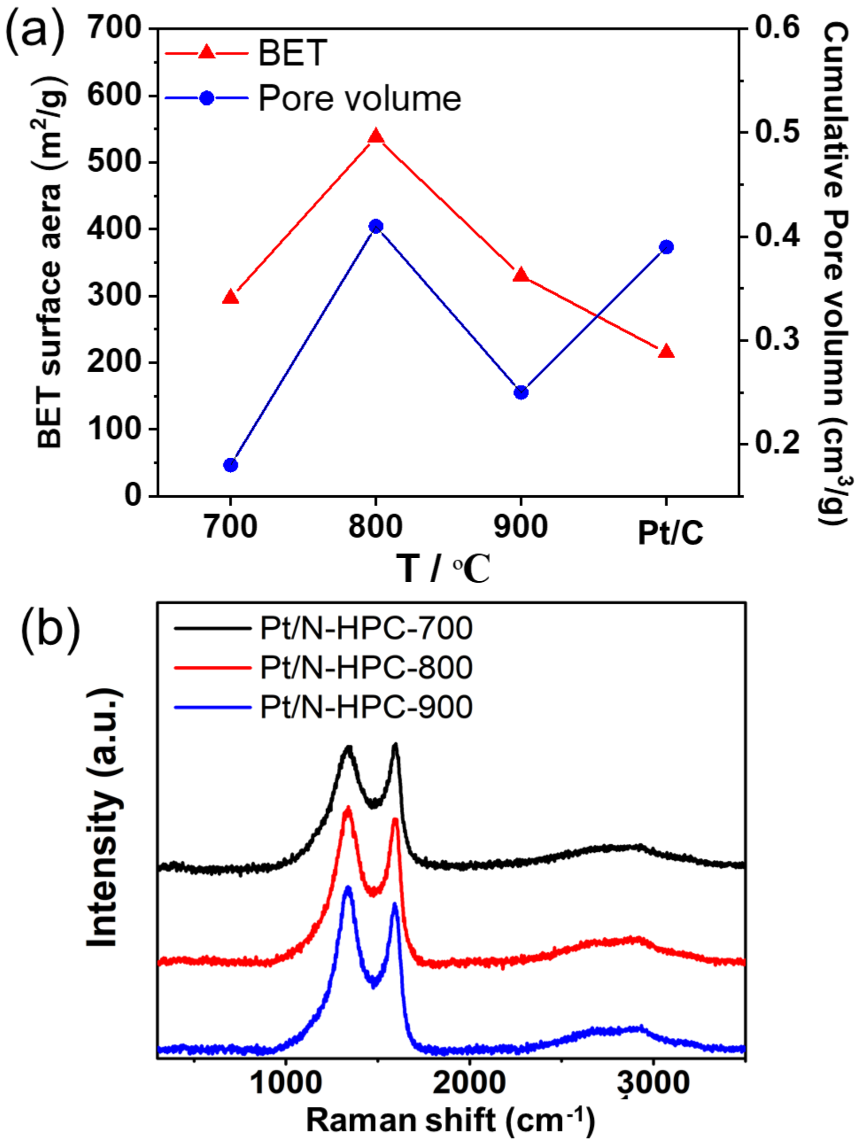

Figure S6. (a) Brunauer–Emmett–Teller (BET) surface areas and cumulative pore volumes of the Pt/C and Pt/NHPC-T catalysts (T = 700, 800, and 900 °C). (b) Raman spectrum of Pt/NHPC-T (T = 700, 800, 900 ℃).

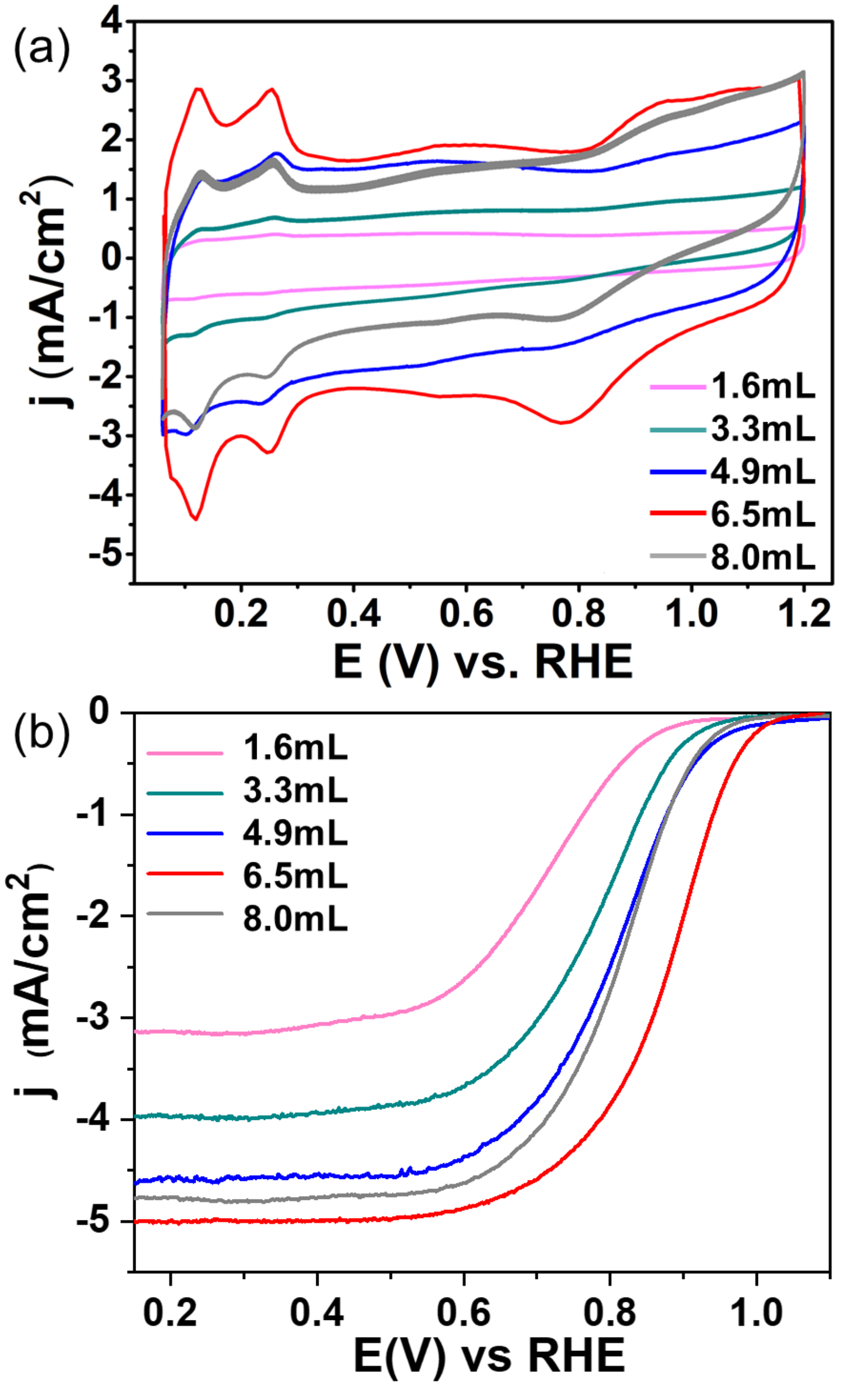

Figure S7. Representative ORR performance measurement (a) Cyclic voltammetry (CV) data for x-Pt/NHPC-800 (x = 1.6 mL, 3.3 mL, 4.9 mL, 6.5 mL, 8.0 mL) in N_2-saturated 0.1 M $HClO_4$. (b) RDE polarization curves of x-Pt/NHPC-800 (x = 1.6 mL, 3.3 mL, 4.9 mL, 6.5 mL, 8.0 mL) in O_2-saturated 0.1 M $HClO_4$ with a scan rate of 10 mV s^{-1} and rotation speed of 1,600 rpm.

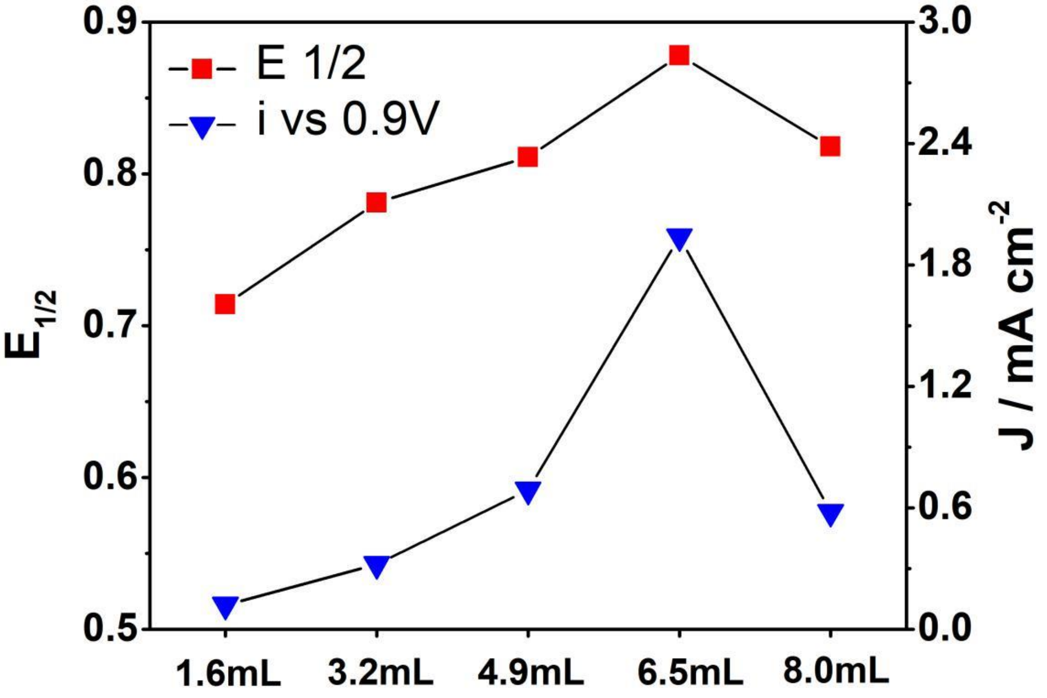

Figure S8. Half-wave potential and electric current density at 0.9 V versus RHE for x-Pt/NHPC-800 (x =1.6 mL, 3.3 mL, 4.9 mL, 6.5 mL, 8.0 mL).

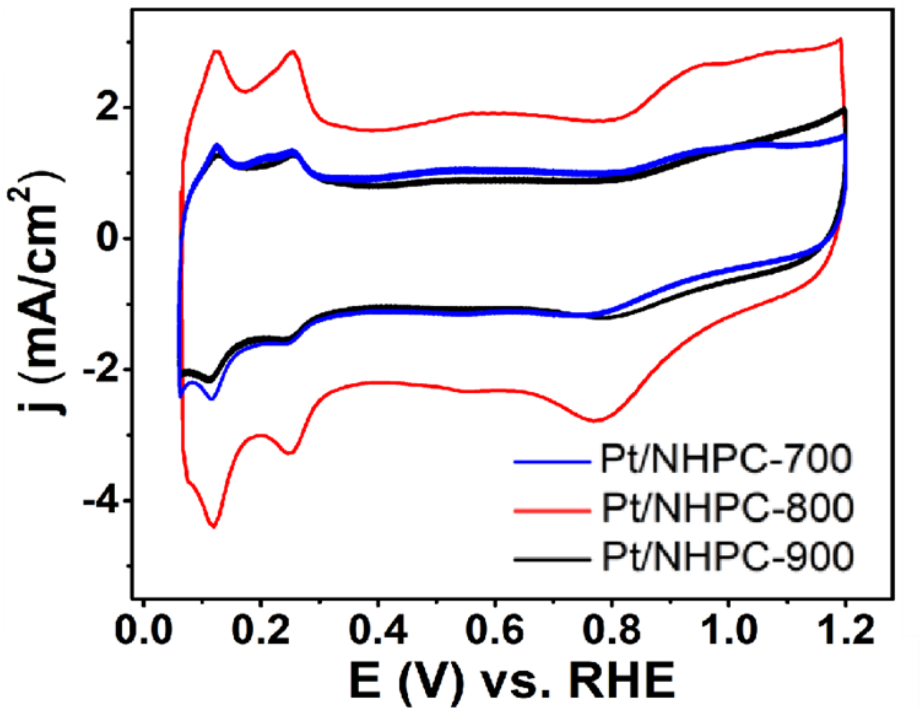

Figure S9. The cyclic voltammetry (CV) data for Pt/NHPC-T (T = 700, 800, 900 °C) in N_2-saturated 0.1 M $HClO_4$.

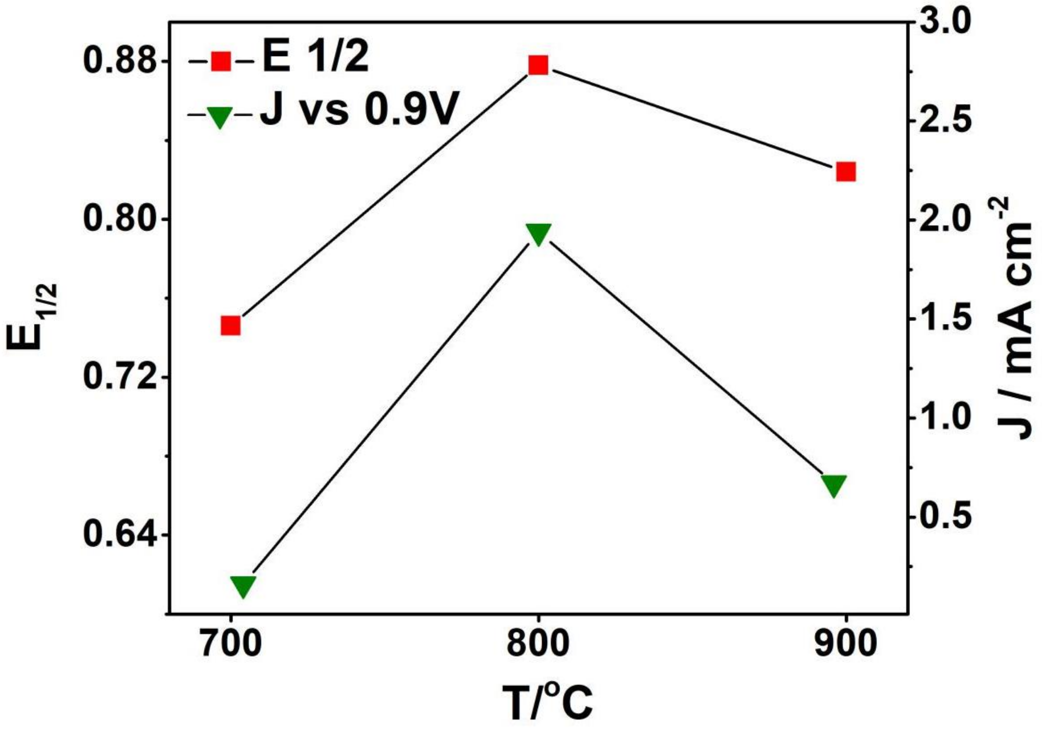

Figure S10. Half-wave potential and electric current density at 0.9 V versus RHE for the serial samples of Pt/NHPC-T (T = 700, 800, 900 ℃).

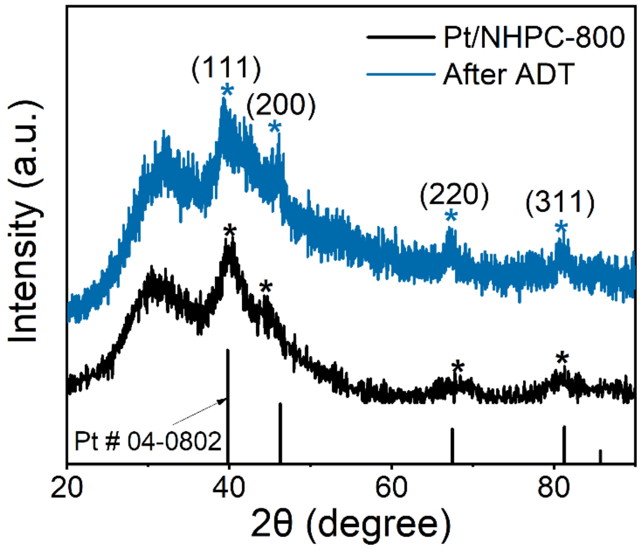

Figure S11. XRD pattern of Pt/NHPC-800 before and after ADT.

Table S1. Surface composition of Pt/NHPC-700, Pt/NHPC-800, Pt/NHPC-900, calculated from XPS results.

Sample	Species concentration (wt %)			
	C	O	N	Pt
Pt/NHPC-700	75.31	11.14	2.01	11.27
Pt/NHPC-800	71.54	7.83	3.15	17.48
Pt/NHPC-900	71.12	8.83	1.83	18.23
Pt/NHPC-800-ADT	67.75	13.98	3.35	14.92

Table S2. Distribution of each Pt species among Pt/NHPC-700, Pt/NHPC-800, Pt/NHPC-900, Pt/C, and Pt@resin precursors, collected from fitting the Pt 4f XPS spectra results (normalized to the surface Pt atoms of each material).

Sample	Species concentration (at. %)		
	Pt (0)	Pt (II)	Pt (IV)
Pt/NHPC-700	20.8	9.9	69.3
Pt/NHPC-800	62.8	26.8	10.4
Pt/NHPC-900	66.8	22.4	10.8
Pt@resin	-	-	100
Pt/C	55.7	33.8	10.5
Pt/NHPC-800-ADT	51.5	33.9	14.6

Table S3. The electrochemical performance of the as-prepared Pt/NHPC-700, Pt/NHPC-800, Pt/NHPC-900, and commercial Pt/C.

Sample	ECSA (m^2 g^{-1})	Specific activity (mA cm^{-2})	Mass activity (A mg_{Pt}^{-1})	$E_{1/2}$ (V)
Pt/NHPC-700	16.9	0.077	0.013	0.746
Pt/NHPC-800	55.2	0.300	0.165	0.878
Pt/NHPC-900	15.8	0.240	0.038	0.824
Pt/C	96.0	0.111	0.107	0.856
Pt/NHPC-800 After ADT	54.6	0.247	0.135	0.870
Pt/C After ADT	65.4	0.092	0.061	0.841
